# Fear avoidance beliefs in back pain-free subjects are reflected by amygdala-cingulate responses

**DOI:** 10.3389/fnhum.2015.00424

**Published:** 2015-07-24

**Authors:** Michael L. Meier, Phillipp Stämpfli, Andrea Vrana, Barry K. Humphreys, Erich Seifritz, Sabina Hotz-Boendermaker

**Affiliations:** ^1^Balgrist University HospitalZurich, Switzerland; ^2^Center of Dental Medicine, University of ZurichZurich, Switzerland; ^3^Department of Psychiatry, Psychotherapy and Psychosomatics, Hospital of Psychiatry, University of ZurichZurich, Switzerland; ^4^MR-Center of the Psychiatric Hospital and Department of Child and Adolescent Psychiatry, University of ZurichZurich, Switzerland

**Keywords:** fear avoidance, low back pain, amygdala, pgACC, vulnerability, fMRI, PPI analysis, chronic low back pain

## Abstract

In most individuals suffering from chronic low back pain, psychosocial factors, specifically fear avoidance beliefs (FABs), play central roles in the absence of identifiable organic pathology. On a neurobiological level, encouraging research has shown brain system correlates of somatic and psychological factors during the transition from (sub) acute to chronic low back pain. The characterization of brain imaging signatures in pain-free individuals before any injury will be of high importance regarding the identification of relevant networks for low back pain (LBP) vulnerability. Fear-avoidance beliefs serve as strong predictors of disability and chronification in LBP and current research indicates that back pain related FABs already exist in the general and pain-free population. Therefore, we aimed at investigating possible differential neural functioning between high- and low fear-avoidant individuals in the general population using functional magnetic resonance imaging. Results revealed that pain-free individuals without a history of chronic pain episodes could be differentiated in amygdala activity and connectivity to the pregenual anterior cingulate cortex by their level of back pain related FABs. These results shed new light on brain networks underlying psychological factors that may become relevant for enhanced disability in a future LBP episode.

## Introduction

Acute low back pain (LBP) has a favorable prognosis; most patients recover within 6 weeks (Koes et al., 2001). However, a small minority of patients develop disabling persistent and/or recurrent LBP that accounts for a considerable burden in terms of pain and suffering, loss of productivity and substantial health care expenditures (Bronfort et al., 2004; Rapoport et al., 2004; Peterson et al., 2012). In the development of chronic disability and in the absence of identifiable organic pathology, psychosocial variables, specifically fear avoidance beliefs (FABs), have been recognized as significant prognostic factors (Vlaeyen and Linton, 2000; Buchbinder et al., 2001; Buer and Linton, 2002; Leeuw et al., 2007b; Chou and Shekelle, 2010; Wertli et al., 2013). Such fears are represented by a subjectively misinterpreted importance of back pain and an associated vulnerability of the spine and can lead to avoidance behavior due to fear that certain behaviors will worsen the pain. Excessive FABs result in heightened disability and are an obstacle for recovery from acute, subacute, and chronic LBP (Rainville et al., 2011).

From a neurobiological perspective, the characterization of brain imaging signatures in pain-free individuals before any injury will be crucial if we are to identify the relevant networks for (back) pain chronicity and associated disability (Dunn et al., 2013; Denk et al., 2014). Recent research has narrowed the characterization of brain signatures involved in each stage of the disease and in the transition from (sub-) acute to chronic LBP (Baliki et al., 2012). Hashmi et al. (2013) have convincingly shown that during the transition from acute to chronic back pain, brain activity related to the perception of back pain shifted from regions linked to nociception to brain networks associated with emotion. In addition to these functional abnormalities, structural abnormalities, such as white matter abnormalities, seem to play an important role during the chronification process (Mansour et al., 2013). Patients have shown decreases in gray matter density in areas associated with pain processing (Ivo et al., 2013; Lloyd et al., 2014). At the stage of chronic LBP, these brain changes can show discriminative power leading to the ability to distinguish between individuals with and without chronic LBP on a neural level (Callan et al., 2014; Ung et al., 2014).

However, the mechanisms underlying these brain changes are still unclear. Although FABs have found broad empirical support as a predictor of chronic LBP and associated disability, neural substrates of the fear component could not yet be demonstrated when comparing chronic LBP patients and pain-free controls (Barke et al., 2012). Regarding pain-free controls, there is evidence that back pain related FABs already exist in the healthy population (Buer and Linton, 2002). Therefore, we aimed at investigating possible differential neural functioning between high- and low fear-avoidant individuals in the general population using functional magnetic resonance imaging (fMRI). Risk assessment was carried out by means of the adapted version of the Tampa Scale for Kinesiophobia (TSK for the general population, TSK-G) questionnaire. The TSK-G measures FABs in the general population by specifically focusing on movement-related fear of pain (Houben et al., 2005). During the fMRI measurements, subjects observed randomly presented video clips of daily activities that have been described as being perceived as either harmful or harmless for the back (Leeuw et al., 2007a).

On the neural level, we primarily focused on amygdala activity and connectivity because this brain area represents a key region in the evaluation and representation of fear intensity and pain and in deciphering threats in visual scenes (Kryklywy et al., 2013). Furthermore, amygdala activity and its functional connectivity to the prefrontal cortex (PFC) have been shown to be meaningfully different between individuals with different emotional modulation strategies and are strongly related to treatment response in chronic pain conditions (Bushnell et al., 2013; Silvers et al., 2014; Simons et al., 2014b). We hypothesized that there would be differential amygdala activity and task-related connectivity in individuals with high TSK-G scores compared to those with low TSK-G scores. Such a finding could reflect brain network properties underlying psychological factors that may become relevant for enhanced disability in a future LBP episode.

## Materials and Methods

### Subjects and Questionnaires

Following an online advertisement, 28 healthy subjects (15 females, mean age = 29.73, SD = 10.4) completed a modified German 17-item version of the TSK-G^1^ (Houben et al., 2005). The TSK-G consists of a 4-point Likert scale ranging from “strongly disagree” to “strongly agree” and includes questions such as “If I had LBP and I were to try to overcome it, my pain would increase.” The questionnaire was originally validated in a Dutch sample of 2240 individuals divided in two groups of people with and without back complaints. Psychometric research indicated a sufficient reliability (Cronbach’s α = 0.78) and high Tampa Scale for Kinesiophobia-G scores predicted pain catastrophising, pain intensity, pain-related health indices. Thus, the authors recommended the use of the TSK-G as a measure of FABs in general population studies (Houben et al., 2005). Furthermore, to control for possible influence of general anxiety, all subjects completed the State and Trait Anxiety Inventory (STAI) which is a common questionnaire that measures state and trait anxiety levels (Spielberger, 1971). Exclusion criteria were acute and/or recurrent back pain within the last 6 months, past chronic pain episodes, and a history of psychiatric or neurological disorders. Two subjects were excluded due to excessive head movements (>2.5 mm) during MR data acquisition, leaving a total sample size of 26 subjects for the final analysis. All subjects provided written informed consent for the participation in the experiment. The study was approved by the local ethics committee (Zurich, Switzerland) and was conducted in accordance with the Declaration of Helsinki.

For the statistical analyses, subjects were divided into groups with high and low TSK-G scores. TSK-G subgroups were defined using a median split (median = 35), which resulted in 13 TSK-G_low_ and 13 TSK-G_high_ scorers. Groups were age- and gender-matched (Mann–Whitney *U*-test for age: *p* = 0.10; chi-square test for gender: *p* = 0.31).

### Scanning Parameters

All measurements were performed on a 3-T whole-body MRI system (Philips Achieva, Best, the Netherlands), equipped with a 32-element receiving head coil and MultiTransmit parallel RF transmission. Each imaging session consisted of a survey scan, a B1 calibration scan (for MultiTransmit), a SENSE reference scan and a high resolution T1-weighted anatomical scan. fMRI data were acquired with whole-brain gradient-echo EPI sequences (365 volumes), consisting of 37 slices in the axial direction with the following parameters: field of view = 240 mm × 240 mm; acquisition matrix = 96 × 96; slice thickness = 2.8 mm; interleaved slice acquisition; no slice gap; TR = 2100 ms; TE = 30 ms; SENSE factor = 2.5; flip angel 80°. Anatomical data were obtained with a 3D T1-weighted turbo field echo scan consisting of 145 slices in sagittal orientation with the following parameters: field of view = 230 mm × 226 mm; slice thickness = 1.2 mm; acquisition matrix = 208 × 203; repetition time = 6.8 ms; echo time = 3.1 ms; flip angle = 9° number of signal averages = 1.

### Experimental Protocol

**FIGURE 1:**
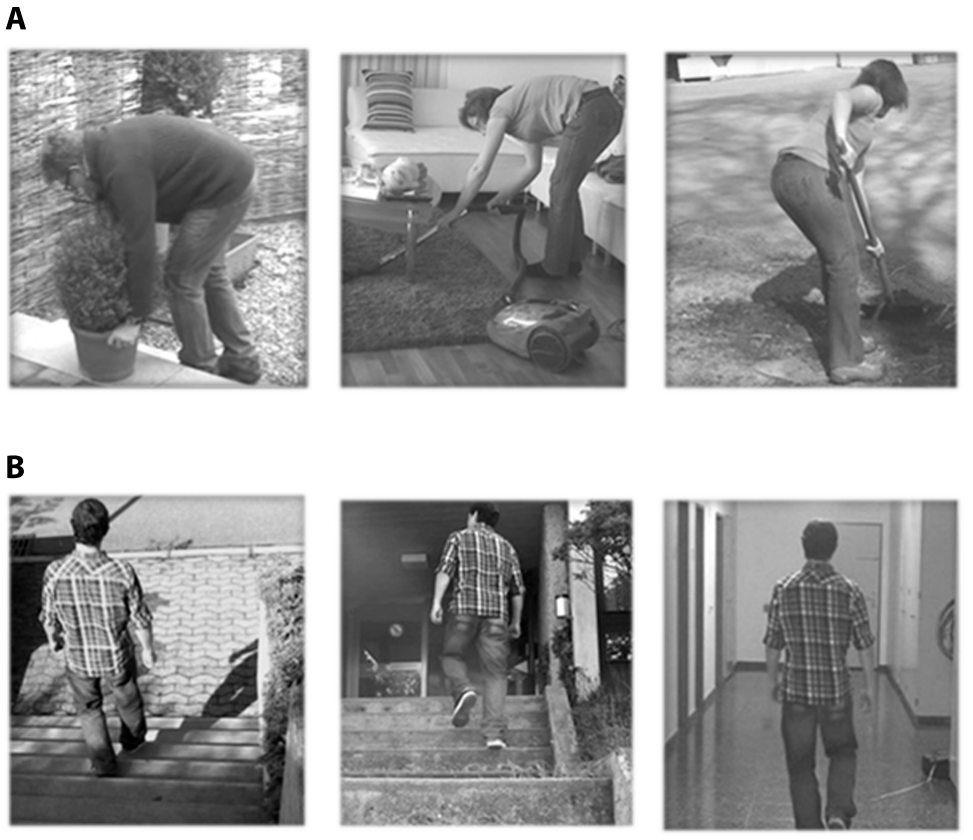
**Types of video clips.** **(A)** Harmful daily activities: shoveling soil with bent back, lifting a flowerpot with slightly bent back and vacuum cleaning under a coffee table with bent back. **(B)** Harmless activities: walking up and down the stairs and walking on even ground.

The stimuli consisted of video clips with a duration of 4 s that showed potentially harmful activities for the back (shoveling soil with a bent back, lifting a flowerpot with slightly bent back and vacuum cleaning under a coffee table with a bent back) and harmless activities (walking up and down the stairs and walking on even ground). The videos were recorded from a third person perspective (**Figure 1**) and standardized in terms of duration of the potentially aversive movements. These daily activities were selected from the short electronic version of the Photograph Series of Daily Activities that has established a fear hierarchy of daily activities based on ratings of perceived harmfulness (Leeuw et al., 2007a). The video clips were displayed using MR-compatible goggles (Resonance Technology, Northridge, USA) connected to a computer running Presentation^®^ software (Neurobehavioral Systems, Davis, CA, USA). Subjects were asked to carefully observe the video clips, which were shown in pseudo-randomized order (no more than two identical consecutive trials). The fMRI measurement with a total duration of ∼8 min consisted of one fMRI session including 30 trials, and the three harmful and harmless activities were each presented five times. Immediately after the observation of the video clips, participants were asked to rate the perceived harmfulness of the activity on a visual analog scale (VAS). The VAS was anchored with the endpoints “not harmful at all” (0) and “extremely harmful” (10) and was shown for 4 s. All ratings were performed using a MR compatible track ball (Current Desings, Philadelphia, PA, USA) that moved the indicator on the VAS scale. The duration of the inter-stimulus interval (ISI, after the VAS rating, black screen with a green fixation cross) was jittered between 6 and 8 s and. The ISI was considered as a baseline, although there is no inherent baseline associated with the blood oxygen-level-dependent (BOLD) signal (Stark and Squire, 2001). We assumed that the baseline represented something akin to a zero-activity condition which was compared with activity during the different tasks.

### Image Preprocessing and Event-Related Analysis

Except the preceding five dummy scans, all functional scans were included in the final analysis. Functional EPI volumes of each subject were corrected for differences in head motion, spatially normalized according to the Montreal Neurological Institute (MNI) space and finally smoothed with a 8 mm full-width at half-maximum (FWHM) Gaussian kernel. To control for confounding head movement effects, individual movement parameters (translations in *x*, *y* and *z*-direction, as well as rotations around *x*, *y*, and *z* axis) were implemented in the first level model as regressors of no interest. Excessive head motion was defined as a dislocation of more than once the in-plane voxel resolution (>2.5 mm). For removing the low frequency noise, a high-pass filter with a cut-off of 128 s was used. Trials were modeled as boxcar regressors and convolved with the standard canonical hemodynamic response function (HRF) as implemented in SPM8. For the first level analysis, the general linear model (GLM) was fitted for each subject by a design matrix composed of the onsets and duration (4 s) of the pooled harmful and harmless video clips. For each subject, parameter estimates (beta) and contrast images (cons) were computed.

The resulting images were analyzed using a random-effects model to allow for population inferences (Friston et al., 1999). For the between group analyses, two-sample *t*-test were used, as implemented in SPM8. The variance between groups was assumed to be unequal. Error covariance components were estimated using restricted maximum likelihood, as implemented in SPM8. Activations and deactivations associated with the video clips were tested by simple positive and negative *t*-contrasts. Study independent amygdala masks were taken from the probabilistic Harvard-Oxford Cortical and Subcortical Structural Atlas^2^ The probability threshold for belonging to the respective brain region was set to *p* >0.5. To control for false positives within the whole-brain results, we used cluster-based family-wise error correction (FWE) based on the Gaussian Random Field Theory (Chumbley and Friston, 2009). The identified clusters were considered to be significant if they fell below a cluster-corrected *p*(FWE) > 0.05. The resulting corrected SPM maps were extracted with MarsBaR^3^, color-coded and superimposed onto the MNI single-subject-T1 brain using MRIcroGL^4^ For percent signal change computations, rfxplot^5^ was used, and correlations were analyzed using Spearman’s rank correlation coefficient (Glascher, 2009).

### Functional Connectivity

The main advantage of psycho-physiological-interactions (PPIs) analysis is that it assesses co-variance between regions across time, and therefore provides a test of task effects on connectivity. For each subject, we extracted the deconvolved time course averaged across the bilateral amygdala clusters (identified by means of the amygdala probability masks, see above) of the contrast “harmful activities > baseline,” pooled across groups (the statistically more rigorous contrast “harmful activities > harmless activities did not reveal any significant results). Subsequently, separate psychological terms (harmful and harmless video clips), physiological regressors (time course of seed region) and PPI interaction terms, as well as the movement parameters, were included in a generalized PPI model. The generalized form of the context-dependent PPI approach increases the flexibility of the statistical modeling and improves single-subject model-fit, thereby increasing the sensitivity to true positive findings and a reduction in false positives (McLaren et al., 2012). The resulting PPI connectivity estimates were then taken into a factorial design, as implemented in SPM8. Whole-brain functional connectivity analysis was performed using the bilateral amygdala cluster as a seed. Identified clusters were considered to be significant when falling below a cluster-corrected *p*(FWE) < 0.05 (cluster extend was 65 voxels). An independent region of interest (ROI) for the pregenual anterior cingulate cortex (pgACC) was created using the MNI peak (4 40 12) with a 6 mm sphere reported in Loggia et al. (2013). Correlations between brain activity and TSK-G scores were analyzed using Spearman’s rank correlation coefficient.

## Results

### Behavioral Results

The overall mean TSK-G score (*N* = 26) was 35.38 (SD = 1.52). TSK-G scores ranged from 22 to 57 points. The mean score of the TSK-G_high_ group (*N* = 13) was 41.38 (SD = 1.56) and for TSK-G_low_ (*N* = 13) 29.38 (SD = 1.01). The VAS ratings were analyzed using a repeated-measures ANOVA with within-subject factor “video type” and between-subject factor “group.” A significant effect of “video type” could be observed [*F*(1,24) = 10.74, *p* = 0.04] whereas neither an effect of “group” [*F*(1,24) = 2.54, *p* = 0.12] nor an interaction effect “group × video type” could be detected [*F*(1,24) = 0.31, *p* = 0.58]. The *post hoc t*-test revealed that the potentially harmful activities were rated as more hazardous than the harmless activities [*t*(25) = 9.98, *p* = 0.01, mean rating harmful = 4.81, SD = 1.8, mean rating harmless = 1.2, SD = 1.2]. The overall mean state score was 43.19 (SD = 4.18) whereas the mean trait score was 42.80 (SD = 3.24). Importantly, state and trait anxiety levels did not differ between the high and low TSK-G groups [Mean scores: TSK-G_high_ group: state (43.38, SD = 4.73) trait (43.46, SD = 3.38)/TSK-G_low_ group: state (43.00, SD = 3.74) trait (42.15, SD = 3.10)/Two sample *t*-test, state: *t*(24) = 0.23, *p* = 0.82; trait: *t*(24) = 1.03, *p* = 0.32]. Moreover, state scores did not correlate with TSK-G scores (Pearson’s *r* = -0.47, *p* = 0.82) whereas trait scores showed a statistical trend (Pearson’s *r* = 0.34, *p* = 0.09, uncorrected). These findings indicate a distinct and state-trait anxiety independent role of movement-related fear of pain as assessed by the TSK-G questionnaire.

### Functional Imaging Results

**FIGURE 2:**
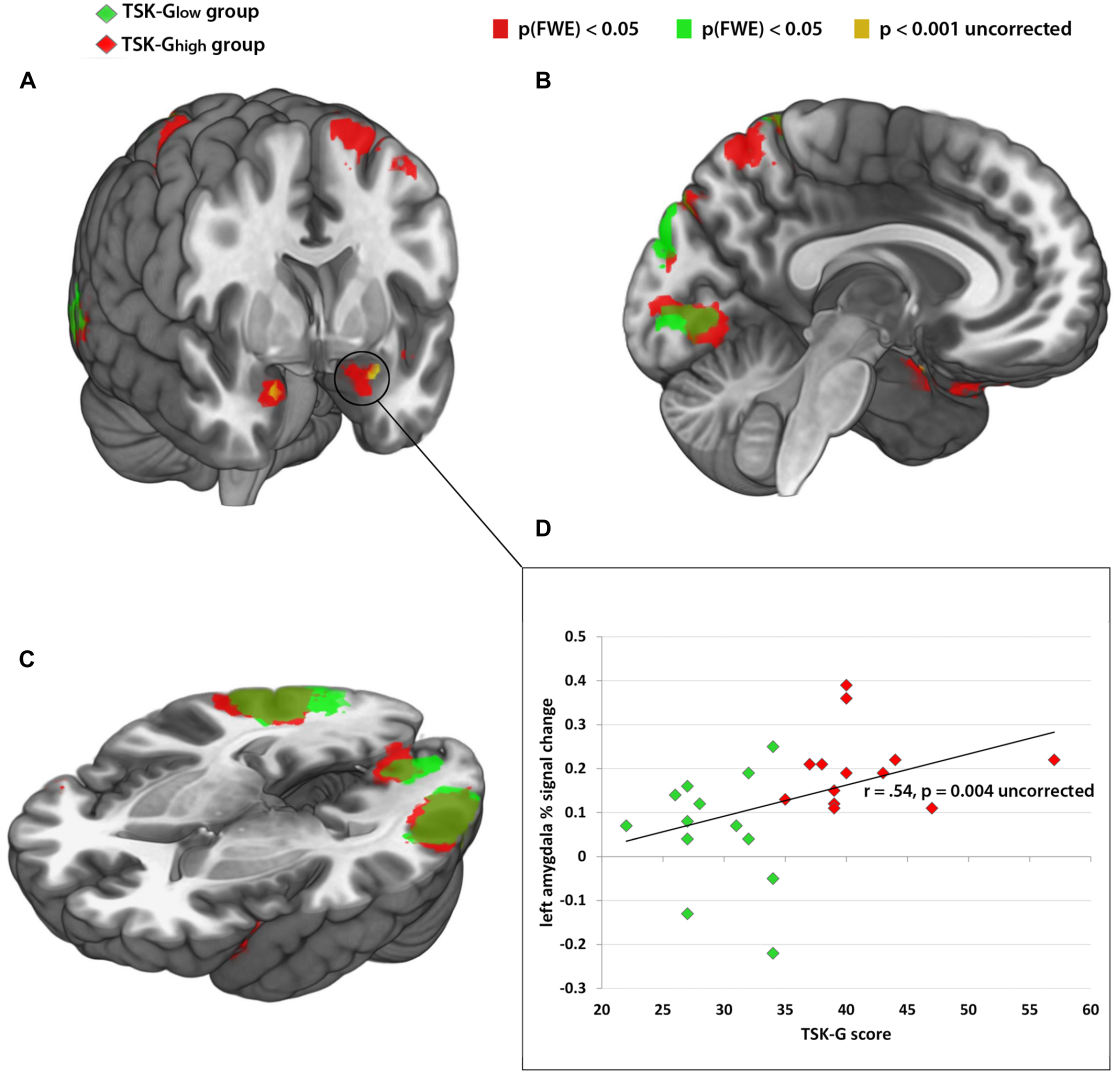
**(A–C)** Red color: brain activity of TSK-G_high_ group from the contrast “harmful activities > baseline,” *p*(FWE) > 0.05. Green color: brain activity of TSK-G_low_ group from the contrast “harmful activities > baseline,” *p*(FWE) > 0.05. Yellow color: enhanced brain activity of the TSK-G_high_ group compared to the TSK-G_low_ group of the contrast “harmful activities > harmless activities” *p* > 0.001, uncorrected. **(D)** Correlational analysis between left amygdala activity (% signal change) and TSK scores. Red dots indicate TSK-G_high_ scorers, green dots indicate TSK-G_low_ scorers.

The categorical whole-brain analysis of the contrast “harmful activities > harmless activities” yielded significant bilateral amygdala activity in the TSK-G_high_ group, whereas no amygdala activity was observed in the TSK-G_low_ group (peak MNI coordinates: left amygdala -22 -2 -24, right amygdala: 20 0 -22, *p*(FWE) < 0.05, **Table 1A,B**; **Figures 2A–C**). A direct whole-brain comparison (two-sample *t*-test) of the contrast “harmful activities harmless activities” between TSK-G_high_ and TSK-G_low_ groups revealed bilateral activity exclusively in the amygdala (*p* < 0.001, uncorrected, **Figure 2A**; **Table 1C,D**). Furthermore, left amygdala responses (% signal change) to the harmful activities relative to baseline positively correlated with the TSK-G score (*r* = 0.54, *p* = 0.004, uncorrected, **Figure 2D**). No significant correlations were detected for the right amygdala or for harmless activities (all *p*-values > 0.19). Importantly, no significant relationships were detected between left and right amygdala activity and individual state (both *p*-values > 0.1) and trait scores (both *p*-values > 0.7).

**Table 1:** Cluster maxima of the event-related analysis (all clusters are listed here, minimum cluster size 10 voxels, height threshold = *p* < 0.001, uncorrected), clusters which survived multiple comparisons family-wise error correction (FWE) are depicted in bold, MNI, Montreal Neurological Institute.

Cluster size	*p*(FWE)	T	MNI coordinates			Brain region (AAL label)
			*x*	*y*	*z*	
**(A) TSK_high_ “harmful activities < harmless activities”**						
6258	**<0.01**	7.76	-26	-52	72	Left superior parietal lobe (Parietal_Sup_L)
750	**<0.01**	7.94	54	26	18	Right frontal lobe (Frontal_Inf_Tri_R)
1696	**<0.01**	7.37	58	-54	0	Right middle temporal gyrus (Temporal_Mid_R)
595	**<0.01**	6.46	-20	0	68	Left superior frontal gyrus (Frontal_Sup_L)
417	**<0.01**	6.24	-26	20	-24	Left inferior frontal gyrus (Frontal_Inf_Orb_L)
885	**<0.01**	6.00	24	-54	62	Right superior parietal lobe (Parietal_Sup_R)
144	**<0.01**	5.32	20	0	-22	Right parahippocampal gyrus (ParaHippocampal_R)
124	**<0.01**	5.66	-22	-2	-24	Left amygdala (Amygdala_L)
51	<0.08	5.16	30	-30	-8	Right hippocampus (Hippocampus_R)
148	**<0.01**	5.13	-56	14	24	Left inferior frontal gyrus (Frontal_Inf_Oper_L)
83	**<0.05**	4.57	16	-26	38	Right precentral gyrus
47	<0.2	4.30	66	-18	32	Right supramarginal gyrus (SupraMarginal_R)
23	<0.8	4.29	2	-2	38	Right cingulate gyrus (Cingulum_Mid_R)
37	<0.1	4.76	2	62	16	Right medial frontal gyrus (Frontal_Sup_Medial_R)
52	<0.8	4.35	62	-36	8	Right superior temporal gyrus (Temporal_Sup_R)
55	<0.2	4.52	-6	56	34	Left medial frontal gyrus (Frontal_Sup_Medial_L)
23	<0.5	3.96	24	-76	46	Right superior parietal lobe (Occipital_Sup_R)
**(B) TSK_low_ “harmful activities > harmless activities”**						
2016	**<0.01**	8.42	-48	-26	34	Left post-central gyrus (SupraMarginal_L)
683	**<0.01**	6.35	24	-54	62	Right superior parietal lobe (Parietal_Sup_R)
3663	**<0.01**	5.94	-50	-74	-2	Left inferior temporal gyrus (Occipital_Mid_L)
21	<0.7	5.55	-28	-28	-8	Left hippocampus (Hippocampus_L)
85	**<0.02**	5.23	22	-84	46	Right superior occipital gyrus (Cuneus_R)
57	<0.5	4.60	54	22	10	Right inferior frontal gyrus (Frontal_Inf_Tri_R)
77	<0.1	4.41	64	-36	20	Right supramarginal gyrus (Temporal_Sup_R)
37	<0.5	4.18	50	-26	36	Right post-central gyrus (SupraMarginal_R)
28	<0.8	4.12	16	-96	4	Right cuneus (Calcarine_R)
**(C) TSK_low_ > TSK_high_ “harmful activities > harmless activities”**						
No significant clusters						
**(D) TSK_high_** > **TSK_low_ “harmful activities** > **harmless activities”**						
13	<0.1	4.35	-26	2	-20	Left amygdala (Amygdala_L)
16	<0.1	4.03	20	4	-24	Right amygdala (Amygdala_R)

### Functional Connectivity Results

**FIGURE 3:**
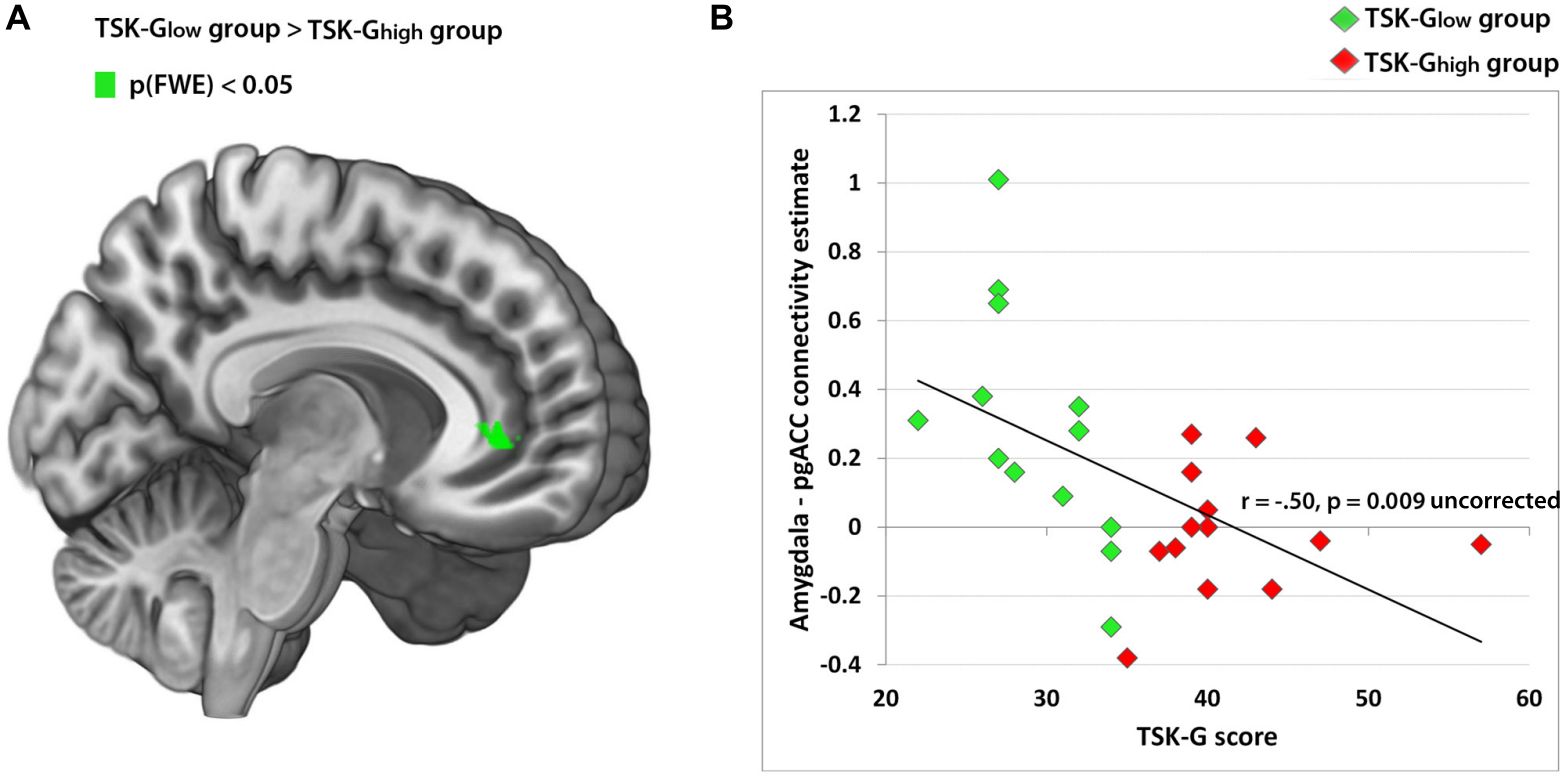
**(A)** Amygdala-pgACC functional connectivity cluster (TSK-G_low_ > TSK-G_high_ from the contrast “harmful activities > baseline”), peak MNI 12 38 0, *p*(FWE) < 0.05. **(B)** Correlation analysis of amygdala-pgACC (Loggia et al., 2013; pgACC peak) functional connectivity and TSK scores. Red dots indicate TSK-G_high_ scorers, green dots indicate TSK-G_low_ scorers.

Using the amygdala as a seed, the whole-brain analysis of the contrast “harmful activities > baseline” revealed no significant enhanced connectivity of the TSK-G_high_ group compared to the TSK-G_low_ group. However, the reverse comparison (TSK-G_low_ > TSK-G_high_) yielded a significant cluster in the pgACC [Peak MNI 12 38 0, *p*(FWE) < 0.05, **Table 2**; **Figure 3A**]. The respective correlation analysis using the study-independent pgACC ROI (Loggia et al., 2013) revealed a significant and negative relationship between the amygdala/pgACC functional coupling strength and the TSK-G score (*r* = -0.50, *p* = 0.009, uncorrected, **Figure 3B**). Further correlational analyses of connectivity estimates between these two regions did not show significant correlations between the contrast “harmless activities > baseline” and TSK-G scores or the state and trait scores (all *p*-values > 0.11).

**Table 2:** Cluster maxima of the functional connectivity analysis with the bilateral amygdala as a seed (all clusters are listed here, minimum cluster size 10 voxels, height threshold = *p*< 0.001, uncorrected, clusters which survived multiple comparisons FWE are depicted in bold), MNI, Montreal Neurological Institute.

Cluster size	*p*(FWE)	T	MNI coordinates (mm)			Brain region (AAL label)
			**x**	**y**	**z**	
**(A) TSK _high_ “harmful activites > baseline”**
217	**<0.01**	7.98	18	-8	-22	Right parahippocampal gyrus (ParaHippocampal_R)
291	**<0.01**	5.98	38	-64	20	Right middle temporal gyrus
131	**<0.01**	5.70	38	-46	-14	Right fusiform gyrus (Fusiform_R)
12	<0.1	5.52	20	20	28	Right frontal lobe
137	**<0.01**	5.36	-20	-6	-22	Left hippocampus (Hippocampus_L)
32	<0.4	5.04	-36	-66	-4	Left occipital lobe
50	<0.1	4.47	-46	-62	16	Left middle occipital gyrus (Temporal_Mid_L)
16	<0.1	4.38	-18	-46	0	Left precuneus (Precuneus_L)
10	<0.1	4.33	-48	-80	14	Left inferior occipital gyrus (Occipital_Mid_L)
41	<0.2	4.22	-62	-28	44	Left post-central gyrus
12	<0.1	4.22	-30	-18	-20	Left hippocampus (Hippocampus_L)
18	<0.9	4.03	56	-48	6	Right middle temporal gyrus (Temporal_Mid_R)
14	<0.1	3.99	-6	-32	-6	Left brain stem
12	<0.1	3.94	-4	-56	-4	Left cerebellum (Cerebelum_4_5_L)
**(B) TSK_low_ “harmful activites> baseline”**
160	**<0.01**	6.64	18	-6	-20	Right parahippocampal gyrus (ParaHippocampal_R)
597	**<0.01**	6.42	-6	36	18	Left anterior cingulate (Cingulum_Ant_L)
165	**<0.01**	5.16	6	36	2	Right anterior cingulate (Cingulum_Ant_R)
32	<0.4	5.08	-12	18	-4	Left caudate (Caudate_L)
180	<**0.01**	5.01	14	-56	22	Right precuneus (Precuneus_R)
79	**<0.01**	4.95	-44	12	-16	Left superior temporal gyrus (Temporal_Pole_Sup_L)
40	<0.20	4.92	52	38	0	Right inferior frontal gyrus (Frontal_Inf_Tri_R)
54	<0.08	4.78	-10	8	-14	Left nucleus accumbens (Olfactory_L)
58	**<0.05**	4.68	56	24	2	Right inferior frontal gyrus (Frontal_Inf_Tri_R)
31	<0.5	4.40	-42	16	2	Left insula (Insula_L)
27	<0.6	4.37	-54	8	38	Left middle frontal gyrus (Precentral_L)
26	<0.6	4.33	-44	-82	20	Left middle occipital gyrus (Occipital_Mid_L)
35	<0.3	4.31	54	-66	22	Right middle temporal gyrus (Temporal_Mid_R)
15	<0.1	4.26	56	14	22	Right inferior frontal gyrus (Frontal_Inf_Oper_R)
12	<0.1	4.24	6	8	72	Right superior frontal gyrus (Supp_Motor_Area_R)
20	<0.8	4.15	-56	10	24	Left inferior frontal gyrus (Frontal_Inf_Oper_L)
10	<0.9	4.10	10	6	-16	Right lateral front-orbital gyrus
11	<0.9	4.09	46	20	-12	Right superior temporal gyrus (Frontal_Inf_Orb_R)
13	<0.9	4.04	0	12	46	Left medial frontal gyrus (Supp_Motor_Area_L)
10	<0.9	3.93	-52	-26	28	Left inferior parietal lobe (SupraMarginal_L)
**(C) TSK_high_ > TSK_low_ “harmful activites > baseline”**
No significant clusters						
**(D) TSK_low_ > TSK_high_ “harmful activites > baseline”**
65	**<0.03**	6.02	12	38	0	Right anterior cingulate (Cingulum_Ant_R)
51	<0.1	5.32	-6	36	18	Left anterior cingulate (Cingulum_Ant_L)
24	<0.7	4.63	-28	54	16	Left middle frontal gyrus (Front_Mid_L)
20	<0.8	4.43	44	26	0	Left inferior frontal gyrus (Frontal_Inf_Tri_R)
11	<0.9	4.23	-12	18	-2	Left caudate (Caudate_L)
14	<0.9	4.17	10	24	44	Right medial frontal gyrus (Frontal_Sup_Medial_R)
24	<0.7	4.02	-42	8	-12	Left insula (Insula_L)

## Discussion

Personality and psychosocial environment factors seem to play an important role in the variability in individual resilience to dysfunctional alterations in the presence of pain. Specific psychological processes and underlying brain networks may be involved in conferring vulnerability to painful conditions that likely contribute to some of these discrepancies (Denk et al., 2014; Simons et al., 2014a).The most consistent finding is the strong predictive power of FABs and pain-related fear for perceived disability in chronic pain (Vlaeyen et al., 1995a,b; Cook et al., 2006). Although not everyone agrees that pain-related fear should be considered as a phobia, pain researchers and clinicians alike do agree on the importance of pain-related fear and FABs in explaining disabilities and the transition from acute to chronic musculoskeletal pain (Vlaeyen et al., 1995a,b; Vlaeyen and Linton, 2000; Houben et al., 2005; Wertli et al., 2013). However, no neural substrates of back pain related FABs have been found to date (Barke et al., 2012). The results of the current study demonstrate that the observation of potentially harmful daily activities induced enhanced amygdala activity in pain-free individuals without a history of chronic LBP episodes but with elevated fear of movement, as measured by the TSK-G questionnaire. The functional connectivity analysis yielded a reduced functional amygdala – pgACC coupling in TSK-G_high_ scorers.

Event-related and connectivity measures showed significant, although divergent, correlations between the brain activity induced by the observation of potentially harmful daily activities and TSK-G scores. Enhanced amygdala activity was positively correlated with TSK-G scores, whereas stronger amygdala-pgACC coupling was negatively associated with TSK-G scores. Importantly, neither state anxiety nor trait anxiety scores showed a significant relationship with brain activity or functional connectivity. These findings demonstrate the stimulus-specific role of task-related amygdala reactivity and movement-related fear of pain.

Increased affective responses involve bottom–up emotional processes that are mediated by subcortical structures, amongst which the amygdala plays a key role (Seifritz et al., 2003; Wager et al., 2004; Ellingsen et al., 2013; Denk et al., 2014; Silvers et al., 2014). The amygdala activation observed in the current study is consistent with models that implicate this brain region in the evaluation and representation of perceived fear intensity and pain, as well as contributing to the deciphering of threats in visual scenes (Goossens et al., 2007; Brugger et al., 2011; Kryklywy et al., 2013). Furthermore, accumulating evidence points to the amygdala as an important site for a reciprocal relationship between persistent pain and negative affective states such as fear and anxiety (Neugebauer et al., 2004). Likewise, the elicitation of negative affect and pain consistently activates the pgACC (Shackman et al., 2011). The amygdala maintains intensive crosstalk with forebrain regions, such as the anterior cingulate cortex (ACC) and prefrontal regions (e.g., pgACC, vmPFC, and the dorsolateral prefrontal cortex, DLPFC); this crosstalk forms a neural circuit involved in emotional control and modulation of pain within the descending pain modulatory system (DPMS; Bushnell et al., 2013; Denk et al., 2014). The DPMS constitutes a powerful neural circuit that regulates nociceptive processing in the dorsal horn of the spinal cord and thereby controls which signals enter the brain. As such, it plays an important role in modulating the eventual pain experience (Tracey and Dickenson, 2012; Denk et al., 2014).

Furthermore, there is unique evidence for the critical role of the pgACC in pain inhibition forming an anti-nociceptive network involving subcortical structures such as the amygdala and the periaqueductal gray (PAG; Petrovic et al., 2002; Wager et al., 2004; Bingel et al., 2006; Eippert et al., 2009; Loggia et al., 2013). Previous studies demonstrated that placebo analgesia increases functional coupling between the pgACC and subcortical structures such as the amygdala (Ellingsen et al., 2013). Furthermore, pgACC connectivity with the default-mode network (DMN) has been associated with pain-protective effects (Loggia et al., 2013). Hence, the observed stimulus specific hyperreactivity of the amygdala, and its reduced functional coupling to the pgACC, in individuals with elevated FABs might indicate a premorbid neural mechanism in a pain-free state. This finding may confer a vulnerability of TSK-G_high_ scorers to a possible transition from acute to chronic state in future LBP episodes.

Although differences on a neural level were demonstrated, both groups rated the potential harmfulness of activities equivalently. In addition to a possible lack of sensitivity of the applied VAS scale, the absence of group differences on a behavioral level may indicate an unconscious neural process. This theory is in line with investigations that have demonstrated that the amygdala and medial prefrontal brain regions can be activated and exhibit automatic responses that are outside the focus of attention and conscious processes (Vuilleumier et al., 2002; Bryant et al., 2008). Furthermore, it has been proposed that this network provides a rapid and automatic alerting mechanism for responding to unconscious signals of fear that is “vital for the automatic orienting of attention toward the stimulus and to highlight the stimulus for further cognitive evaluation” (Liddell et al., 2005; Bryant et al., 2008).

However, whether the origin of these alterations in TSK-G_high_ scorers are either inherent or maladaptive remains elusive (Denk et al., 2014). In the general population, large surveys have shown that a range of erroneous back pain-related FABs exist that have arisen from social means, including fear inducing information, and vicarious exposure, such as observations, regardless of the presence of back pain (Buchbinder et al., 2001, 2008; Gross et al., 2006). On the neural level, Phelps et al. (2001) have demonstrated that fears acquired through verbal instruction were associated with robust activation in the amygdala. Interestingly, this activation was predominantly observed in the left amygdala, and thus corresponds to the results of the current study, where the TSK-G scores were only significantly correlated with activity in the left amygdala. These results indicate a left amygdalar dominance of back pain-related fears, which might be acquired through social and/or instructed fear learning. Further support for a left amygdalar dominance in the physiological expression of instructed fear learning comes from subjects with unilateral amygdala damage (Olsson and Phelps, 2007). However, further studies on instructed fear are needed to confirm this left dominance of the amygdala.

A growing body of evidence points to psychological factors, in particular FABs that serve as strong predictors of disability and chronification in LBP. The current investigation provides initial evidence for a correlative neurobiological substrate that might reflect premorbid brain network properties underlying psychological factors that may become relevant for enhanced disability in a future LBP episode. This finding may lead to a more mechanistic understanding on the neural level that supports preventative psychology-based interventions in acute episodes or in exposure *in vivo* in individuals with elevated FABs. Furthermore, there seem to be a call for greater public education in terms of erroneous beliefs regarding back pain. However, the current findings have to be carefully interpreted. A detailed understanding whether changes in these brain signatures are causal in producing cLBP is currently far impossible as it relies on the detailed knowledge of biological and environmental subject variables (Denk et al., 2014). Further studies based on long-term observations including patients are necessary to establish a causal relationship between the indicated brain signatures as a vulnerability factor for chronic LBP.

## Conflict of Interest Statement

The authors declare that the research was conducted in the absence of any commercial or financial relationships that could be construed as a potential conflict of interest.

## Footnotes

1 http://onlinelibrary.wiley.com/doi/10.1016/S1090-3801(09)60838-7/pdf

2 http://www.fmrib.ox.ac.uk/fsl

3 http://marsbar.sourceforge.net/

4 http://www.cabiatl.com/mricrogl/

5 http://rfxplot.sourceforge.net/
